# Imaging transplanted stem cells in real time using an MRI dual-contrast method

**DOI:** 10.1038/srep13628

**Published:** 2015-09-02

**Authors:** Ethel J. Ngen, Lee Wang, Yoshinori Kato, Balaji Krishnamachary, Wenlian Zhu, Nishant Gandhi, Barbara Smith, Michael Armour, John Wong, Kathleen Gabrielson, Dmitri Artemov

**Affiliations:** 1 The In vivo Cellular and Molecular Imaging Center, Division of Cancer Imaging Research, The Russell H. Morgan Department of Radiology and Radiological Sciences, The Johns Hopkins University School of Medicine, Baltimore, MD21205, USA; 2 The Sidney Kimmel Comprehensive Cancer Center, The Johns Hopkins University School of Medicine, Baltimore, MD21205, USA; 3 The Department of Radiation Oncology and Molecular Radiation Sciences, The Johns Hopkins University School of Medicine, Baltimore, MD21287, USA; 4 The Institute for Basic Biomedical Sciences, The Johns Hopkins University School of Medicine, Baltimore, MD21205, USA; 5 The Department of Molecular and Comparative Pathobiology, The Johns Hopkins University School of Medicine, Baltimore, MD21205, USA

## Abstract

Stem cell therapies are currently being investigated for the repair of brain injuries. Although exogenous stem cell labelling with superparamagnetic iron oxide nanoparticles (SPIONs) prior to transplantation provides a means to noninvasively monitor stem cell transplantation by magnetic resonance imaging (MRI), monitoring cell death is still a challenge. Here, we investigate the feasibility of using an MRI dual-contrast technique to detect cell delivery, cell migration and cell death after stem cell transplantation. Human mesenchymal stem cells were dual labelled with SPIONs and gadolinium-based chelates (GdDTPA). The viability, proliferation rate, and differentiation potential of the labelled cells were then evaluated. The feasibility of this MRI technique to distinguish between live and dead cells was next evaluated using MRI phantoms, and *in vivo* using both immune-competent and immune-deficient mice, following the induction of brain injury in the mice. All results were validated with bioluminescence imaging. In live cells, a negative (T_2_/T_2_*) MRI contrast predominates, and is used to track cell delivery and cell migration. Upon cell death, a diffused positive (T_1_) MRI contrast is generated in the vicinity of the dead cells, and serves as an imaging marker for cell death. Ultimately, this technique could be used to manage stem cell therapies.

Stem cell therapies are currently being investigated, both pre-clinically and clinically, for the repair of brain injuries and a variety of neurodegenerative disorders^1^,^2^. A major obstacle to the clinical translation of these therapies has been the inability to noninvasively evaluate the administration of proper cell doses, while ensuring the survival and biological functioning of the transplanted stem cells^3^,^4^. Consequently, there is a need for the development of non-invasive imaging techniques capable of monitoring the delivery, survival, engraftment, migration, and distribution of transplanted stem cells *in vivo,* with high spatial and temporal resolution^5^.

Currently, SPECT imaging of indium-111-oxine-labelled cells is the only FDA-approved method for tracking transplanted stem cells^6^,^7^. However, SPECT imaging agents have shorter half-lives compared to MRI agents, and this significantly limits their application for the long-term monitoring of transplanted stem cells^8^. Additionally, like most imaging modalities that employ exogenous cell labelling with imaging probes, it is difficult to report on the survival of transplanted cells^9^.

Magnetic resonance imaging (MRI) provides several advantages over radionuclide imaging for monitoring stem cell therapies. These include: superior delineation of morphology; no exposure to radiation; and the possibility of monitoring transplanted cells over long periods of time^10^,^11^,^12^,^13^. Although exogenous stem cell labelling with superparamagnetic iron oxide nanoparticles prior to stem cell transplantation is currently the most employed cell labelling method in both preclinical and clinical trials^14^,^15^,^16^,^17^,^18^,^19^,^20^, monitoring cell death following transplantation is still a challenge^21^,^22^,^23^. Consequently, this is currently an area of active research^24^,^25^,^26^,^27^,^28^,^29^,^30^,^31^,^32^,^33^,^34^,^35^,^36^,^37^.

In this study, we evaluated the feasibility of detecting in real-time, cell delivery, cell migration and cell death of transplanted stem cells, using an MRI dual-contrast technique, and validated the findings with bioluminescence imaging (BLI). The MRI dual-contrast technique exploits the differences in contrast generation mechanisms and diffusion coefficients between two different classes of contrast agents, to detect cell migration and cell death. The technique employs slow-diffusing, superparamagnetic iron oxide nanoparticles (SPIONs) and fast-diffusing, gadolinium-based chelates^38^,^39^. Whereas SPIONs generate a signal loss (negative, T_2_/T_2_* contrast), the gadolinium chelates generate a signal gain (positive, T_1_ contrast) in the tissue containing them^40^.

We hypothesized that, in live cells, where both contrast agents are entrapped in confined cellular spaces and remain in close proximity to each other, a strong T_2_/T_2_* contrast would be generated by the labelled cells. The T_1_ contrast of the gadolinium chelates in the labelled cells would be quenched^38^,^39^,^41^. Upon cell death, the plasma membranes of the transplanted cells would be breached^42^. The small-sized, fast-diffusing, gadolinium chelates would then diffuse away from the slow-diffusing SPIONs and generate a diffused T_1_ contrast enhancement in the vicinity of the dead cells (Fig. 1). This dynamic T_1_ contrast enhancement in the vicinity of the transplanted cells would then serve as a local imaging marker for cell death. The different MRI signatures (T_2_/T_2_* and T_1_) would be distinguishable using an MRI spin echo pulse sequence with appropriate acquisition parameters. Based on our previous studies, we determined that it is possible to separate both T_2_/T_2_* and T_1_ signals using appropriate acquisition parameters, when both agents are as little as ~15 μm away from each other^38^,^39^.

The feasibility of this technique to detect, cell delivery, cell migration and cell death was evaluated *in vitro* using MRI phantoms and *in vivo* using an image-guided, radiation-induced murine model of brain injury, in both immune-competent and immune-deficient mice.

## Results

### Dual magnetic stem cell labelling and assessment of its biological effects

In order to detect the presence of human mesenchymal stem cells by MRI, cells were dual magnetically labelled in the absence of a transfection agent, using commercially available contrast agents. Bionized nanoferrite (BNF) nanoparticles and gadolinium-diethylenetriaminepentaacetate (GdDTPA) were used as SPIONs and gadolinium chelates, respectively. Since the production and commercialization of the clinically-approved SPIO (Feridex) was discontinued in 2009, bionized nanoferrites (BNFs) were chosen for the following reasons: 1) their high magnetization and magnetization saturation (49 emu/g iron and >79 emu/g iron respectively); 2) their optimum size (80 nm); 3) their negative charge; 4) their optimum iron content (10 mg/mL); 5) their biocompatible surface coating (starch); and 6) their commercial availability.

**Figure 2:**
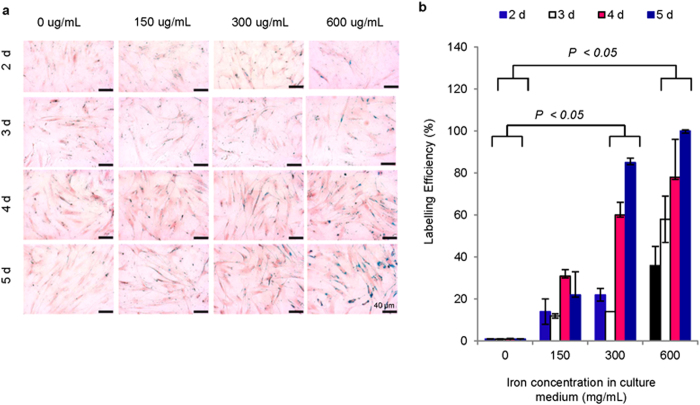
Dual magnetic stem cell labelling. (**a**) Perl’s Prussian blue (PPB) staining of stem cells labelled with various concentrations of BNF nanoparticles for various periods of time, followed by incubation with GdDTPA (30 mM) for 18 h. (**b**) BNF labelling efficiency in dual magnetically labelled cells determined by light microscopy (n = 3; *P* < 0.05).

**Figure 3:**
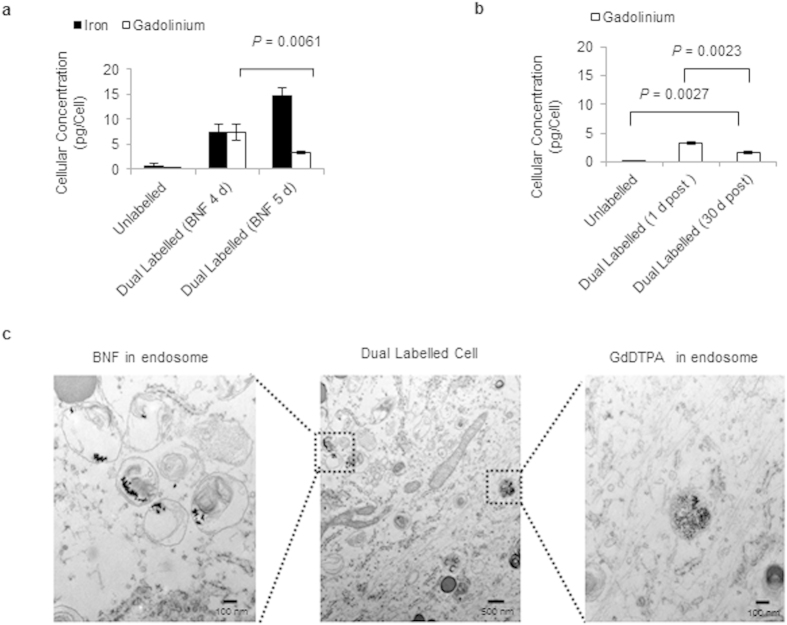
Cellular iron and gadolinium concentrations and sub-cellular localization of both contrast agents. (**a**) Cellular iron and gadolinium concentrations in dual magnetically labelled stem cells, 24 h post labelling. These results suggest that the cellular uptake of BNF nanoparticles does, indeed, affect the cellular uptake of GdDTPA (n = 3, *P* = 0.0061). (**b**) ICP-MS analysis of dual magnetically labelled cells, 30 days post labelling, indicates the presence of gadolinium in the cells (n = 3; *P* = 0.0027). (**c**) TEM images indicating the sub-cellular localization of BNF nanoparticles and GdDTPA in dual magnetically labelled cells.

Under the optimum labelling condition determined by Perl’s Prussian Blue staining (Fig. 2), the cells contained 14.8 ± 1.7 pg of iron/cell and 3.3 ± 0.2 pg of gadolinium/cell (Fig. 3a), as determined by inductively coupled plasma mass spectrometry (ICP-MS). Although this labelling protocol did not employ transfection agents generally used in cell labelling, the application of transfection agents did not seem to change the diffusion profile of GdDTPA (Supplementary Fig. S1). Furthermore, the low molecular weight GdDTPA was retained in the cells for at least 30 days (Fig. 3b), as determined by ICP-MS. These findings are comparable to those generally reported for exogenous stem cell labelling for MRI detection^43^,^44^. Transmission electron microscopy (TEM) images showed accumulation of both contrast agents in endocytic vesicles (Fig. 3c), confirming entrapment of the agents in sub-cellular structures with limited water accessibility, and further ensuring T_1_ contrast quenching^45^.

**Figure 4:**
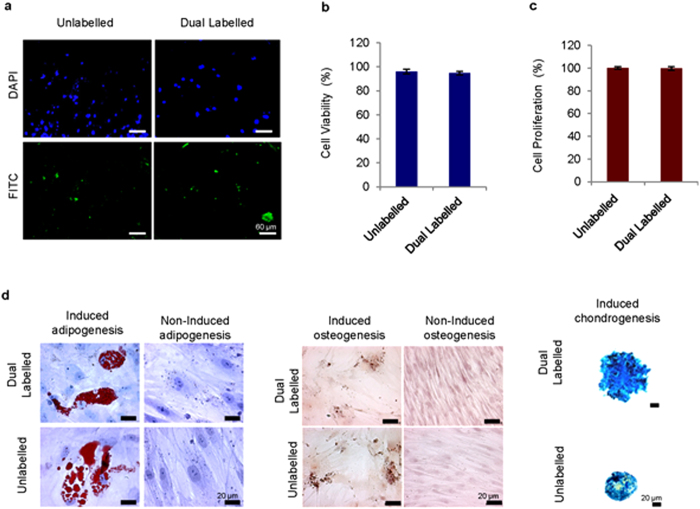
Biological effects of dual magnetic stem cell labelling. (**a**) Apoptosis detection in dual magnetically labelled and unlabelled stem cells, assessed by the TUNEL assay, shows no difference in staining. (**b**) Cell viability of dual magnetically labelled and unlabelled cells, 24 h post labelling, assessed by Trypan Blue assay, show no significant difference between the cells (n = 3). (**c**) Cell proliferation rates of dual magnetically labelled and unlabelled cells, 10 days post labelling, assessed by MTT assay, show no significant difference between the cells (n = 3). (**d**) Multi-lineage differentiation of dual magnetically labelled and unlabelled cells shows no difference in adipogenesis induction (Oil Red O staining); osteogenesis induction (Alizarin Red S staining); or chondrogenesis induction (Alcian Blue staining).

Overall, labelling the cells under the optimum labelling conditions did not affect their viability, proliferation rates or differentiation potential (Fig. 4).

### Phantom imaging of cell death by MRI and BLI

The feasibility of this MRI dual-contrast technique to distinguish between live and dead cells was next evaluated using MRI phantoms, and the results were validated with BLI. Human mesenchymal stem cells were stably transduced to constitutively express the luciferase reporter gene (Supplementary Method S1 and Supplementary Fig. S2) and then dual magnetically labelled as describe above. Phantoms containing luciferase-expressing, dual magnetically labelled live and dead cells, respectively, were then prepared. These phantoms were imaged by both MRI and BLI, one hour following preparation.

**Figure 5:**
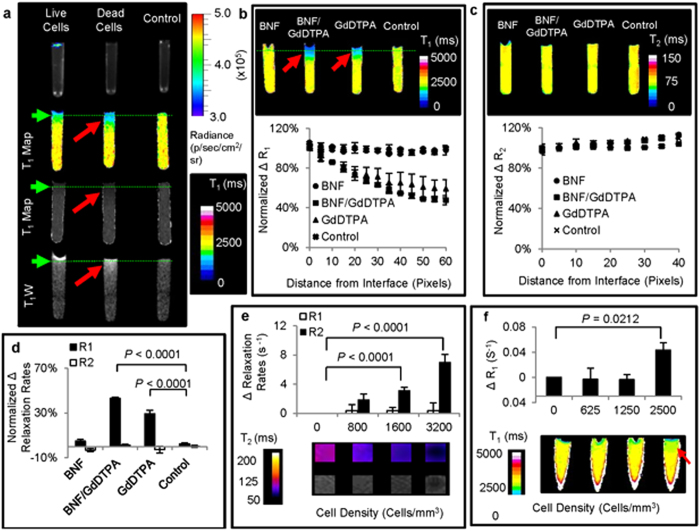
Phantom imaging of cell death detection. (**a**) BLI and corresponding T_1_ maps, and T_1_ images of 2% (w/v) agarose gel, on which were placed respective samples of live and dead luciferase-transduced and dual magnetically labelled cells. (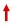) represents diffused T_1_ contrast enhancement in the vicinity of dead dual magnetically labelled cells. (**b**) T_1_ maps of 2**%** (w/v) agarose gel and corresponding longitudinal relaxation rate changes (%ΔR_1_) along the tubes, on which were placed respective dead cell samples (n = 3). (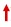) represents diffused T_1_ contrast enhancement in the vicinity of dead dual magnetically labelled cells. (**c**) T_2_ maps of 2**%** (w/v) agarose gel containing the respective dead cell samples, and corresponding transverse relaxation rate changes (%ΔR_2_) along the tubes (n = 3). (**d**) Comparison of longitudinal (ΔR_1_) and transverse (ΔR_2_) relaxation rate changes of 2% (w/v) agarose gel, ~10 pixels from the surface, on which were placed dead cell samples (n = 3, *P* < 0.0001). (**e**) Changes in longitudinal (ΔR_1_) and transverse (ΔR_2_) relaxation rates of _2_% (w/v) agarose gel with varying densities of dual magnetically labelled live cells (n = 3, *P* < 0.0001). (**f**) Changes in longitudinal relaxation rates (ΔR_1_) of 2% (w/v) agarose gel pads, ~10 pixels from the cell placement surfaces (n = 3, *P* < 0.0212). Samples were prepared with varying densities of dual magnetically labelled dead cells to estimate the cell death detection limits. (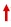) represents T_1_ contrast enhancement in the vicinity of dead dual magnetically labelled cells.

In phantoms containing dead cells, a diffused T_1_ contrast was generated in the vicinity of the cells on T_1_ maps and T_1_-weighted images (Fig. 5a). This suggested the diffusion of GdDTPA, released from the dead cells. Cell death and cell viability in the respective phantoms was confirmed with BLI following MRI (Fig. 5a).

To ensure that the T_1_ contrast generated in the vicinity of the dead dual magnetically labelled cells was indeed the result of GdDTPA released from the dead cells, MRI phantoms of dead dual magnetically labelled cells were imaged and compared to phantoms of dead GdDTPA-labelled and dead BNF-labelled cells. In both dual magnetically labelled and GdDTPA-labelled dead cell phantoms, T_1_ contrast enhancements were observed in the vicinity of the dead cells on T_1_ maps (Fig. 5b) and T_1_-weighted images (Supplementary Fig. S3). No T_1_ contrast enhancement was observed in the vicinity of BNF-labelled dead cells (Fig. 5a and Supplementary Fig. S3). Collectively, these results confirmed that the T_1_ contrast enhancement observed in the vicinity of the dead cells was, indeed, the result of GdDTPA released from the dead cells. At the GdDTPA concentration released, no T_2_ contrast enhancement was generated on T_2_ maps and T_2_-weighted images in the vicinity of dual magnetically labelled dead cells and GdDTPA-labelled dead cells (Fig. 5c and Supplementary Fig. S3). Quantitative comparison of the longitudinal (R_1_) and transverse relaxation rates (R_2_) in the vicinity of the dead cells (Supplementary Fig. S4) showed a significant change (n = 3; *P* < 0.0001) in R_1_ compared to R_2_, in both dual magnetically labelled and GdDTPA-labelled dead cells (Fig. 5d).

Under the optimum labelling conditions, this technique proved useful in detecting the presence of less than 1.6 × 10^3^ live cells/mm^3^ (Fig. 5e) and the death of as few as 2.5 × 10^3^ cells/mm^3^ (Fig. 5f), measured using MRI spin echo pulse sequences.

### Induction and assessment of brain injury in mice

**Figure 6:**
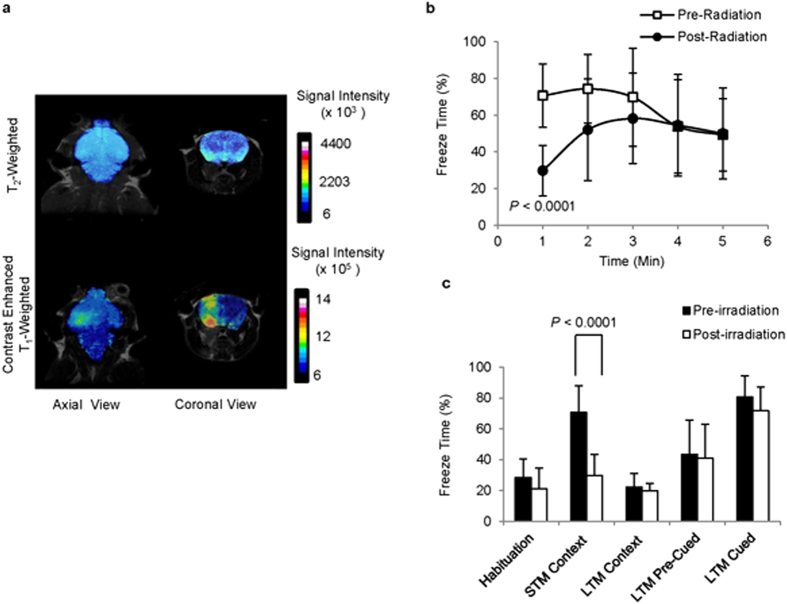
Radiation-induced brain injury in mice. (**a**) T_2_-weighted and contrast-enhanced, T_1_-weighted MR images of a representative irradiated mouse brain, two weeks post irradiation. (**b**) Comparison of changes in the freezing behaviour of mice during the context test session, before and two weeks after brain irradiation (n = 10; *P* < 0.0001). (**c**) Comparison of changes in short-term memory (STM) and long-term memory (LTM), assessed during the context and cued test sessions, before and two weeks after brain irradiation (n = 10; *P* < 0.0001).

The ability of this MRI dual-contrast technique to detect cell death of transplanted stem cells *in vivo* was next evaluated using an image-guided, radiation-induced murine model of brain injury in two groups of mice: severely compromised immune-deficient (SCID) mice of balb/c background; and immune-competent balb/c mice. Following stereotactic focal irradiation (with X-rays of 100 kVp at a dose of 80 Gy and a dose delivery rate of 1.7 Gy/Min (Supplementary Fig. S5)), a change in the permeability of the blood brain barrier of the irradiated brain hemispheres was observed as early as two weeks after irradiation (Fig. 6a) in both groups of mice. This change was clearly visible on contrast-enhanced MR images (Fig. 6a and Supplementary Fig. S6), and corresponded to a change in the short-term memory of the mice, as determined by contextual fear-memory tests (Fig. 6b, c and Supplementary Method S2). Collectively, these changes were used to confirm brain injury.

### *In vivo* imaging of stem cell migration by MRI

Upon establishment of brain injury in the mice, luciferase-transduced and dual magnetically labelled stem cells were stereotactically implanted into the right cerebral hemisphere, contralateral to the radiation-induced lesions in both groups of mice (immune-deficient and immune-competent). The mice were then monitored by both MRI and BLI.

**Figure 7:**
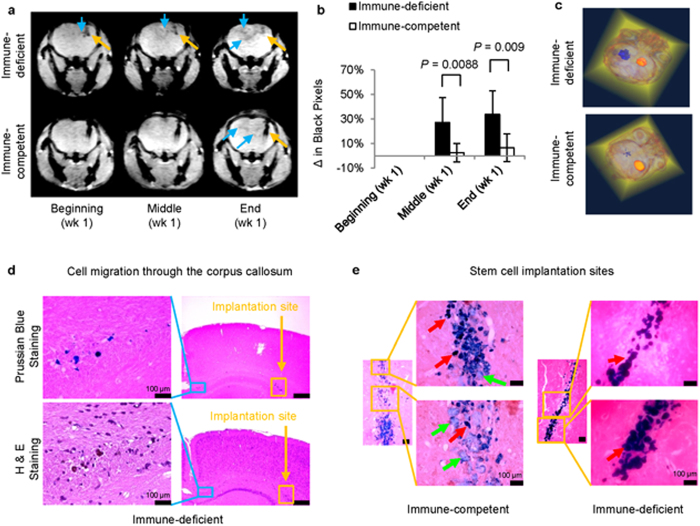
*In vivo* imaging of cell delivery and cell migration. (**a**) T_2_*-weighted images of immune-deficient and immune-competent mouse brains, indicating the site of cell delivery (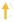) and cell migration to the radiation-induced lesion (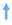). (**b**) Black-pixel analyses of T_2_*-weighted images of mouse brains from both groups at the middle and end of week one, indicating significant cell migration to the radiation-induced lesion site (n = 5; *P* < 0.0088 and *P* < 0.009, respectively). (**c**) Three-dimensional reconstruction of T_2_*-weighted MR images of brains from immune-deficient and immune-competent mice, indicate cell distribution at both the implantation and radiation-induced lesion sites. (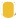) represents the dual magnetically labelled cells lodged at the implantation site. (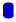) represents the migrated dual magnetically labelled cells at the radiation-induced lesion sites. (**d**) Histology of adjacent slices of a representative immune-deficient mouse brain indicates the migration of iron-containing cells from the implantation site to the lesion site through the corpus callosum. (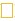) represents the dual magnetically labelled cells lodged at the implantation site. (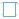) represents migrating dual magnetically labelled cells. (**e**) Perl’s Prussian blue staining of stem cell implantation tracts of both immune-deficient and immune-competent mice. (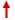) implanted cells, and (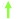) infiltrating cells.

In both groups, following the administration of the cells, there was a significant T_2_* contrast enhancement at the implantation site (Fig. 7a). The feasibility of this technique to image cell migration to the injury site in the contralateral hemisphere was next evaluated by monitoring T_2_* contrast enhancement in the injury site. In both groups, cell migration through the corpus callosum and toward the radiation-induced lesion was observed within the first week of cell implantation (Fig. 7a). This was expected, since cell migration to injuries has been reported to occur as early as 24 h post stem cell transplantation^46^. In immune-competent mice, however, less cell migration was observed, presumably due to early graft rejection^47^. Cell migration was next analysed by calculating the changes in black pixels in the contralateral hemisphere of cell implantation, compared to those in the ipsilateral hemisphere of implantation (Supplementary Methods S3 and Supplementary Fig. S7)^26^. The T_2_* changes in the immune-competent and the immune-deficient mice were compared at the middle and end of week one, using a Kruskal–Wallis one-way analysis of variance, followed by a post-hoc two-sided Scheffé test. The results were considered statistically significant at *P* < 0.05. In immune-deficient mice, black-pixel analyses showed a significant change (n = 5; *P* < 0.0088 and *P* < 0.009, respectively) in the contralateral hemisphere within the first week of implantation (Fig. 7b). In immune-competent mice, however, cell migration was less, presumably due to cell death within the first week^47^.

Three-dimensional reconstructions of high resolution T_2_* images of perfused mouse brains showed the distribution of the transplanted stem cells in both brain hemispheres (Fig. 7c and Supplementary Fig. S8). However, more cells seemed present at the injury site in immune-deficient mice.

Furthermore, histological analyses of the immune-deficient mouse brains confirmed the migration of iron-containing cells through the corpus callosum (Fig. 7d). Additionally, in immune-competent mice, cells of varying morphology which stained positive for iron were observed at the implantation site (Fig. 7e).

### *In vivo* imaging of cell death by MRI and BLI

**Figure 8:**
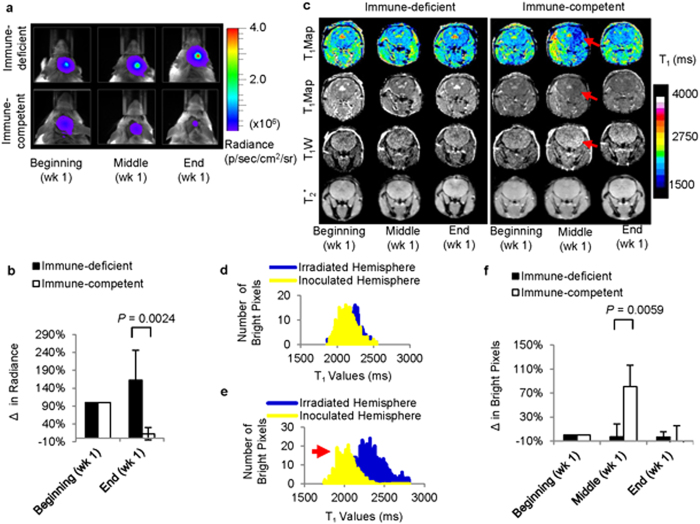
*In vivo* imaging of cell death in real-time. (**a**) Representative bioluminescence images of immune-deficient and immune-competent mice, respectively, within the first week of cell implantation. (**b**) Quantification of radiance in immune-deficient and immune-competent mice, respectively, at the beginning and end of week one. The signals were normalized for each mouse and indicate significant cell death by the end of week one in immune-competent mice (n = 5; *P* < 0.0024). (**c**) Comparison of T_1_ contrast enhancement in immune-deficient and immune-competent mice, respectively, within the first week of cell transplantation. A significant T_1_ contrast enhancement (n = 5; *P* < 0.0059) was observed in the slice adjacent to that of the cell delivery site in immune-competent mice. (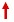) represents T_1_ contrast enhancement in the slice adjacent to that of cell delivery. (**d**) Pixel intensity histograms of the ipsilateral and contralateral hemispheres of cell implantation before graft rejection indicate similar T_1_ values in both hemispheres. (**e**) Pixel intensity histograms of the ipsilateral and contralateral hemispheres of cell implantation after graft rejection indicate lower T_1_ values in the hemispheres ipsilateral to cell implantation. (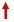) represents T_1_ contrast enhancement in the slice adjacent to that of cell delivery (**f**) Quantification of T_1_ contrast enhancement in immune-deficient and immune-competent mice, respectively, at the beginning, middle, and end of week one. The signals were normalized for each mouse and indicate significant T_1_ contrast enhancement within week one in immune-competent mice (n = 5; *P* < 0.0059).

Following stem cell transplantation in the mice, the viability of the transplanted cells in both groups was monitored by BLI (Fig. 8a). In immune-competent mice, a significant decrease (n = 5; *P* < 0.0024) in radiance (BLI) was observed by the end of week one, compared to that at the beginning of the week (Fig. 8b). Although the cell death of the transplanted cells occurred on different days in the immune-competent group, complete death of the transplanted cells was observed within the first week of transplantation. In immune-deficient mice, however, no significant decrease in radiance was observed by the end of week one, compared to that at the beginning of the week. However, a decrease in radiance was observed in the immune-deficient mice at a later time point (Supplementary Fig. S9).

The feasibility of the MRI dual-contrast technique to detect cell death *in vivo* was evaluated by monitoring T_1_ contrast changes in the vicinity of the cell implantation site. In immune-competent mice, where a decrease in radiance (BLI) was observed within the first week, a significant T_1_ contrast enhancement (n = 5; *P* = 0.0059) was also observed on T_1_-maps in the hemisphere ipsilateral to cell implantation (Fig. 8c). The T_1_ contrast changes were analysed by calculating changes in the bright pixels of the hemisphere ipsilateral to cell implantation, compared to those of the contralateral hemisphere (Supplementary Method S4 and Supplementary Fig. S10), before (Fig. 8d) and after cell death of the transplanted cells (Fig. 8e). By correlating BLI and MRI changes in the immune-competent group (Supplementary Fig. S11), it was estimated that as low as an ~20% decrease in the BLI signal (cell death of ~1.25 × 10^4^ cells) could be detected *in vivo*, based on changes in the T_1_ contrast enhancement. The GdDTPA released from the dead cells was cleared from the system within two days of release (Fig. 8f).

## Discussion

Collectively, these results indicate that the dual MRI contrast technique can be used to monitor the delivery, and migration of dual magnetically labelled transplanted stem cells and also to assess cell death in real-time. Following cell death, the fast-diffusing GdDTPA is released from the breached cells and diffuses away from the slow-diffusing nanoparticles into the surrounding tissues. This generates a characteristic imaging signature with T_1_ contrast enhancement in the vicinity of the dead cells, which serves as a local imaging marker of cell death. In contrast, in live cells, where both contrast agents remain entrapped in confined cellular spaces and in close proximity to each other, no T_1_ contrast enhancement is observed in the cell vicinity.

Although this method shows promise in tracking the initial engraftment, survival, and homing of transplanted therapeutic cells, it has a few limitations. First, due to the rapid clearance of GdDTPA after it is released from dead cells, the imaging schedule is very important in obtaining accurate readings. Second, given the intrinsic low sensitivity of MRI to low molecular weight gadolinium chelates, this method is most suitable for the detection of hyperacute and acute cell death, in which case there is a rapid contrast agent build-up to detectable levels.

Furthermore, although given the small size and rapid diffusion of GdDTPA it should be possible to detect GdDTPA release through the breached membranes of transplanted stem cells, phagocytosed by infiltrating phagocytic immune cells such as macrophages, microglia cells^48^,^49^,^50^,^51^; this method is not suitable for distinguishing between apoptotic and necrotic mechanisms of cell death.

Overall, this study demonstrates the feasibility of noninvasively monitoring in real-time the transplantation, initial engraftment, or rejection of transplanted stem cells, using the MRI dual contrast technique. Since this strategy is based on diffusional differences between a T_2_/T_2_* and a T_1_ contrast agent, it can be applied to a variety of commercially available MRI contrast agents typically used for exogenous stem cell labelling. Ultimately, this technique could be used to manage cell-based therapies by providing timely feedback about if and when cell death of transplanted cells occurs. This could allow the proper tailoring of therapeutic regimens.

## Methods

### Dual magnetic stem cell labeling and assessment of its biological effects

Human mesenchymal stem cells (Lonza Poietics) were cultured in mesenchymal stem cell basal medium (Lonza Poietics), supplemented with 10% mesenchymal stem cell growth supplements, 2% l-glutamine, 0.1% gentamicin, and 0.1% amphotericin. Cells were then dual magnetically labelled with 80 nm carboxylate-surface-modified bionized nanoferrite (BNF) nanoparticles (Micromod Partikeltechnologie GmbH), at 600 μg of iron/mL for five days, and 30 mM of GdDTPA (Magnevist^®^, Shering) for 18 h^45^,^52^. At BNF concentrations above 600 μg of iron/mL, such as 1000 μg of iron/mL, followed by 30 mM of GdDTPA (Magnevist^®^, Shering) for 18 h, a change in the cell morphology was observed (data not shown).

To determine whether the duration of stem cell incubation with BNF nanoparticles affected the cellular uptake of GdDTPA, stem cells that were dual magnetically labelled with 600 μg of Fe/mL for shorter periods of time than the optimum (four days versus five days), followed by GdDTPA for 18 h, were also analysed. The cellular concentration of both iron and gadolinium was assessed one day and 30 days post dual labelling by ICP-MS.

The cell viability, proliferation rates, and apoptosis induction in the labelled cells were next assessed by the Trypan Blue assay (Gibco Laboratories), the MTT assay (Cayman Chemical Company) and the TUNEL assay (Trevegen Inc.), respectively. Following this, the ability of the labelled cells to undergo multi-lineage differentiation into adipocytes, chondrocytes, and osteocytes was determined using differentiation induction media (Lonza Poietics) in accordance with the supplier’s instructions, and the cells were stained appropriately.

### Phantom imaging of cell death by MRI and BLI

In order to evaluate the feasibility of this MRI dual-contrast technique to distinguish between live and dead cells *in vitro*, cells were stably transduced to express the luciferase reporter gene (Supplementary Method S1). Luciferase-expressing cells were then dual magnetically labelled and cell death was induced in a group of cells by subjecting them to three freeze-thaw cycles. Cell death was confirmed by MTT assay. The cells were then placed in 100 μL PCR tubes containing 80 μL of 2% (w/v) agarose gel, topped with 10 μL of cell culture media and imaged by both MRI and BLI, one hour following placement. The dead cell phantoms were imaged in comparison to live cell phantoms maintained under physiological conditions (37 °C and 5% CO_2_) prior to imaging.

Phantom MRI experiments were performed on a 9.4 T Bruker horizontal bore scanner, equipped with a 30 mm radiofrequency coil (Bruker Biospin GmbH). The Paravision 5.1.0 software was used for all image acquisitions. Both T_1_-weighted and T_2_-weighted images were acquired using a spin echo pulse sequence. T_1_-weighted sequence: rapid acquisition with refocused echoes (RARE); echo time (TE) = 7 ms; effective echo time (ETE) = 7; RARE factor = 8; repetition times (TR) = 500, 1000, 2000, 6000 ms; number of averages (NA) = 5; field of view (FOV) = 20 × 40 mm; matrix size (MS) = 128 × 128 pixels; and slice thickness = 0.7 mm. T_2_-weighted sequence: rapid acquisition with refocused echoes (RARE); echo time (TE) = 7 ms; effective echo times (ETE) = 7, 35, 63, 91 ms; RARE factor = 4; repetition time (TR) = 4000 ms; number of averages (NA) = 5; field of view (FOV) = 20 × 40 mm; matrix size (MS) = 128 × 128 pixels; and slice thickness = 0.7 mm. Quantitative pixel-by-pixel reconstruction of T_1_ and T_2_ maps was performed using an in-house program written in IDL software (Exelis Visual Information Solutions). Final image analyses were performed using the NIH ImageJ program.

Phantom BLI experiments were performed using a Xenogen IVIS 200 optical imaging system (PerkinElmer Inc.) and the images were processed using the Xenogen Living Imaging 4.2 software. The radiance from the samples was used to determine cell viability.

### Animal experiments

All animal experimental protocols were approved by the Johns Hopkins University Animal Care and Use Committee. All animal experiments were conducted in accordance with the Johns Hopkins University’s Animal Care and Use Committee guidelines. Four-week-old, male immune-competent (n = 5) and severely compromised immune-deficient (SCID) balb/c mice (n = 5) were purchased from the National Cancer Institute, and used for the experiments. All experimental procedures were carried out under anaesthesia, using a 2% isoflurane, 30% oxygen and air mixture. No blinded or random animal studies were carried out.

### Induction and assessment of brain injury in mice

Brain injury was induced in mice by X-ray irradiation, using a small animal radiation research platform (SARRP)^53^,^54^. Four-week-old mice were anesthetized, and, under the guidance of cone-beam computed tomography (CBTC), a single well-collimated (3 × 3 mm^2^) X-ray beam of 100 kVp was delivered to the left hippocampi at a single dose of 80 Gy and a dose delivery rate of 1.7 Gy/min. Lesion formation and neurological dysfunction in the mice were then monitored by MRI and behavioural studies, respectively.

*In vivo* MRI scans were performed on a 9.4 T Bruker horizontal bore scanner, equipped with a 30 mm radiofrequency coil (Bruker Biospin GmbH). The Paravision 5.1.0 software was used for all image acquisitions. T_2_-weighted images were acquired using a spin echo pulse sequence with the following acquisition parameters. Sequence: rapid acquisition with refocused echoes (RARE); echo time (TE) = 7.6 ms; effective echo time (ETE) = 30.6 ms; RARE factor = 8; repetition time (TR) = 2000 ms; field of view (FOV) = 24 × 22 mm; matrix size (MS) = 256 × 256 pixels; and slice thickness = 0.5 mm. Contrast enhanced T_1_-weighted images were acquired following a bolus intravenous administration of 0.2 mmol/kg of Magnevist^®^ diluted in 0.9% NaCl. T_1_-weighted images were acquired using a spin echo pulse sequence with the following acquisition parameters. Sequence: rapid acquisition with refocused echoes (RARE); echo time (TE) = 7.6 ms; effective echo time (ETE) = 30.6; RARE factor = 8; repetition time (TR) = 500; field of view (FOV) = 24 × 22 mm; matrix size (MS) = 256 × 256 pixels; and slice thickness = 0.5 mm.

Behavioural studies were conducted on all of the mice before irradiation and two weeks after irradiation. Both short-term and long-term memory and learning tests were conducted on all of the mice following a signalled fear-conditioning paradigm: the contextual and cued fear-conditioning tests (Supplementary Methods S2)^55^,^56^. The changes in freezing behaviour before and after irradiation were compared using a Kruskal–Wallis one-way analysis of variance, followed by a post-hoc two-sided Scheffé test. The results were considered statistically significant at *P* < 0.05.

### *In vivo* imaging of stem cell migration and cell death by MRI and BLI

Upon establishment of brain injury in the mice, luciferase-transduced and dual magnetically labelled stem cells (~2.5 × 10^5^ cells in 5 μL) were stereotactically implanted in the right cerebral hemispheres of mice in both groups, contralateral to the radiation-induced lesions. The mice were then monitored with MRI, every day after implantation for the first week, and then, once during the second and third week. Whereas T_2_*-weighted MR images were used to monitor cell delivery and cell migration, T_1_ MRI maps were used to detect cell death. Since, cell death in the immune-competent mice occurred on different days during week one, the days within week one were grouped to facilitate analyses, as follows: Days 1–2 after implantation were grouped as the beginning of week one, days 3–5 after implantation as the middle of week one, and days 6–7 as the end of week one.

All *in vivo* MRI experiments were performed on a 9.4 T Bruker horizontal bore scanner, equipped with a 30 mm radiofrequency coil (Bruker Biospin GmbH). The Paravision 5.1.0 software was used for all image acquisitions. *In vivo* T_2_*-weighted images were acquired using a gradient echo pulse sequence. Sequence: fast low angle shot (FLASH); echo time (TE) = 2 ms; repetition times (TR) = 250 ms; flip angle = 45°; number of averages (NA) = 4; field of view (FOV) = 24 × 24 mm; matrix size (MS) = 128 × 80 pixels; and slice thickness = 0.5 mm. The T_2_* changes in both groups at the middle and end of week one, were compared using a Kruskal–Wallis one-way analysis of variance, followed by a post-hoc two-sided Scheffé test. The results were considered statistically significant at *P* < 0.05.

T_1_-weighted images were acquired using a spin echo pulse sequence. Sequence: rapid acquisition with refocused echoes (RARE); echo time (TE) = 7 ms; RARE factor = 4; repetition times (TR) = 500, 1000, 2000, 6000 ms; number of averages (NA) = 5; field of view (FOV) = 24 × 24 mm; matrix size (MS) = 128 × 128 pixels; and slice thickness = 0.7 mm. Quantitative pixel-by-pixel reconstruction of T_1_ maps was performed using an in-house program written in IDL software (Exelis Visual Information Solutions). Final analyses were performed using the NIH ImageJ program. The T_1_ contrast enhancement during the middle of week one was then compared to that at the beginning of the week, in both the immune-deficient and the immune-competent mouse groups, using a Kruskal–Wallis one-way analysis of variance, followed by a post-hoc individual comparison Scheffé test, based on the Wilcoxon-Mann-Whitney*-*test. The results were considered statistically significant at *P* < 0.05.

All *in vivo* BLI experiments were performed using a Xenogen IVIS 200 optical imaging system (PerkinElmer Inc.), and the images were processed using the Xenogen Living Imaging 4.2 software. The radiance (BLI) from the mice was used to determine cell viability. The radiance at the end of week one was then compared to that at the beginning of the week, in both the immune-deficient and the immune-competent mouse groups, using a Kruskal–Wallis one-way analysis of variance, followed by a post-hoc individual comparison Scheffé test, based on the Wilcoxon-Mann-Whitney*-*test. The results were considered statistically significant at *P* < 0.05.

### *Ex vivo* imaging of stem cell distribution, post-mortem, by MRI

Following the *in vivo* studies, the mice were perfused with 0.01% heparin and 4% paraformaldehyde solution at a rate of 20 mL/min, and further fixed in 4% paraformaldehyde overnight. Intact mouse brains left in the skulls were then immersed in Fomblin LC08 (Ausimont USA Inc.) and scanned by MRI. Post-mortem MRI scans were performed on a 9.4 T Bruker vertical bore scanner, equipped with a 30 mm radiofrequency coil (Bruker Biospin GmbH). The Paravision 5.1.0 software was used for all image acquisitions. Three-dimensional T_2_*-weighted images were acquired with a gradient echo pulse sequence, using the following acquisition parameters. Sequence: Fast low angle shot (FLASH); echo time (TE) = 1.6 ms; repetition times (TR) = 700 ms; flip angle = 45°; number of averages (NA) = 4; field of view (FOV) = 21 × 10 × 12 mm; matrix size (MS) = 132 × 64 × 72 pixel; and slice thickness = 12 mm. The three-dimensional images were then reconstructed using the Amira software.

### Histology

The brains of mice from each group were paraffin-embedded and sectioned into 30 μm slices. Adjacent slices were stained with haematoxylin and eosin for pathological analyses, and with Perl’s Prussian blue stain for the presence of iron. The slides were then analysed by a diagnostic pathologist.

### Statistical analyses

Data is presented as the mean ± standard deviation for at least three independent experiments (technical replicates). Statistical comparisons were made using a Kruskal–Wallis one-way analysis of variance, followed by a post-hoc Scheffé test based on the Wilcoxon-Mann-Whitney*-*test, except when otherwise stated. The results were considered statistically significant at *P* < 0.05.

## Additional Information

**How to cite this article**: Ngen, E. J. *et al.* Imaging transplanted stem cells in real time using an MRI dual-contrast method. *Sci. Rep.* **5**, 13628; doi: 10.1038/srep13628 (2015).

## Supplementary Material

Supplementary Information

## Figures and Tables

**Figure 1:**
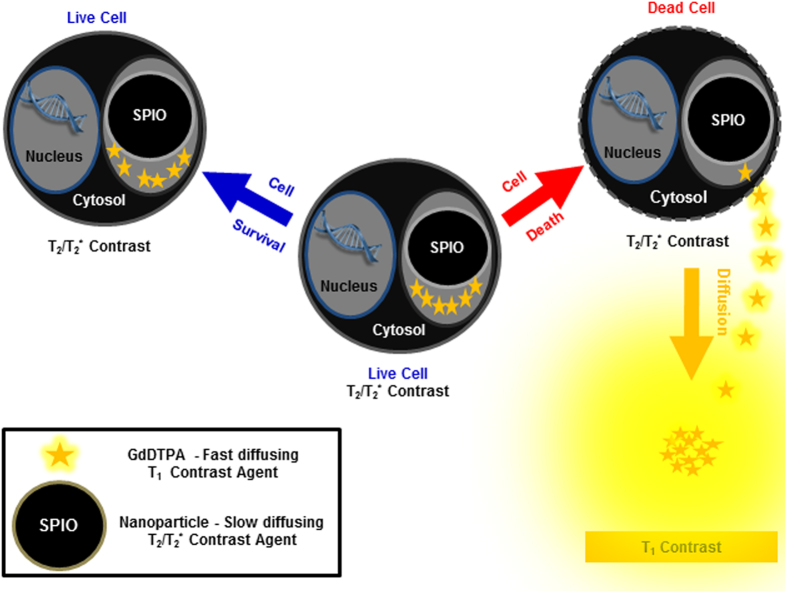
Schematic representing live cell-tracking by T_2_/T_2_* contrast enhancement, and cell death detection by T_1_ contrast enhancement. A diffused T_1_ contrast enhancement is generated in the vicinity of dead cells on T_1_-weighted MR images, and serves as a local imaging marker of cell death. This diffused T_1_ contrast enhancement is not observed in the vicinity of live cells.
