# Supplementary material for: Imaging transplanted stem cells in real time using an MRI dual-contrast method
Source: Sci Rep. 2015 Sep 2;5:13628. doi: 10.1038/srep13628 (PMC4556978; doi:10.1038/srep13628)
Supplement: Supplementary Information [file srep13628-s1.pdf]

Imaging transplanted stem cells in real time using an MRI dual-contrast method

Ethel J. Ngen[1], Lee Wang[1], Yoshinori Kato[1,2], Balaji Krishnamachary[1], Wenlian Zhu[1], Nishant Gandhi[3], Barbara Smith[4], Michael Armour[3], John Wong[3], Kathleen Gabrielson[5], Dmitri Artemov[1,2,*]

[1] The *In vivo* Cellular and Molecular Imaging Center, Division of Cancer Imaging Research, Russell H. Morgan Department of Radiology and Radiological Sciences, The Johns Hopkins University School of Medicine, Baltimore, MD21218, USA.

[2] The Sidney Kimmel Comprehensive Cancer Center, The Johns Hopkins University School of Medicine, Baltimore, MD21218, USA.

[3] The Department of Radiation Oncology and Molecular Radiation Sciences, The Johns Hopkins University School of Medicine, Baltimore, MD21218, USA.

[4] The Institute for Basic Biomedical Sciences, The Johns Hopkins University School of Medicine, Baltimore, MD21218, USA.

[5] The Department of Molecular and Comparative Pathobiology, The Johns Hopkins University School of Medicine, Baltimore, MD21218, USA.

[*]Correspondence should be addressed to: Dmitri Artemov, Ph.D. (Dmitri@mri.jhu.edu)

Supplementary Methods

Supplementary Method S1 ____ 3

Supplementary Method S2 ____ 4

Supplementary Method S3 ____ 5

Supplementary Method S4 ____ 6

Supplementary Figures

Supplementary Figure S1 ____ 7

Supplementary Figure S2 ____ 8

Supplementary Figure S3 ____ 8

Supplementary Figure S4 ____ 9

Supplementary Figure S5 ____ 12

Supplementary Figure S6 ____ 12

Supplementary Figure S7 ____ 13

Supplementary Figure S8 ____ 14

Supplementary Figure S9 ____ 15

Supplementary Figure S10 ____ 16

Supplementary Figure S11 ____ 17

Supplementary Methods

S1. Lentivirus production and stem cell transduction

An infectious viral supernatant was generated by transiently co-transfecting 12 μg of the lentiviral vector containing the gene of interest pHIV-EF-1α-IRES-Luciferase (Addgene), and 6 μg of the packaging vector (pPGKΔR8.2 DVPR), into HEK293T cells. The viral supernatant was collected at 48, 72, and 96 h post transfection, and was then pooled and concentrated using an Amicon Ultra-15 100K cutoff filter (Millipore Billerica). The viral titer was then determined using a p24 ELISA kit (Cell Biolabs Inc.), to detect the HIV-p24 core protein of the vector. Following this, human mesenchymal stem cells were transduced for 24 h with the lentiviral vector, at a multiplicity of infection of 2, using 0.1% polybrene (Sigma-Aldrich) in mesenchymal stem cell growth media (MSCGM), without antibiotics.

To evaluate the correlation between the number of viable cells and the BLI radiance, luciferase-expressing cells were seeded in 10 cm dishes, at a cell density of 5×10^3 cells/cm^2, and grown to 85% confluency. The cells were then harvested, counted, and varying numbers, ranging from 5×10^3 to 4×10^4 cells, were placed in eight-well chamber slides. One hour later, 15 μg/mL of luciferin (PerkinElmer) was added to each well and the plate was read 30 min after luciferin addition (Supplementary Fig. S2).

S2. Assessment of cognitive decline using the signaled fear conditioning paradigm

All procedures were conducted in standard mice operant conditioning chambers (Med Associates Inc.) in accordance with the Johns Hopkins Behavioral Core protocol with a few modifications. Behavioral studies were done in a series of five sessions: an acclimatization session; a conditioning session; a short-term contextual fear memory assessment session; a long-term contextual fear memory assessment session; and a pre-cued and cued testing session.

Briefly, mice were initially placed in the conditioning chambers for five minutes and then returned to their cages. One hour following acclimatization, the mice were returned to the conditioning chambers where a tone (2000 Hz) was played three minutes post placement for 30 s. This was immediately followed by a foot-shock using scrambled alternating current delivered through the grid floor by a constant current shock source (5 mA) for two seconds. The mice were returned to their cages after a total of five minutes. One hour following the conditioning session, the short-term contextual fear memory of the mice was assessed. During this session, the mice were returned to the conditioning chambers for five minutes during which neither tones nor shocks were administered. This was repeated, twenty-four hours following the conditioning session, to assess the long-term contextual fear memory of the mice. One hour following this, the cued fear memory was assessed by re-exposing the mice to the tone (2000 Hz, for three minutes) in a new context and in the absence of a shock. The freezing behavior of the mice during each session was then analyzed.

S3. Black Pixel Analyses

Black pixel analysis was performed, with a few modifications from that described in previous reports. These modifications made the method more robust and reduced the variability observed when calculated using the method described in previous reports.

Briefly, a 2D Z-projection of the minimum signal intensity was generated from the slices of stem cell implantation. Two regions of interest (ROIs) were manually drawn over the ipsilateral hemisphere of stem cell implantation, and the contralateral hemisphere (Supplementary Fig. S7a). Pixel intensity histograms were created for each ROI and a low signal intensity pixel at the base of the distribution of the histogram (ipsilateral hemisphere of stem cell transplantation) was chosen as a threshold (Supplementary Fig. S7b). This threshold corresponded to the pixel intensity of the area around the site of stem cell implantation. The ratio of the number of low signal intensity pixels below the threshold in both hemispheres was then calculated. The value calculated at the first time-point was assigned an arbitrary 0 %. The values calculated at subsequent time-points were normalized relative to the first time-point and reported relative to this value (Supplementary Fig. S7c).

S4. Bright Pixel Analyses

Bright pixel intensity calculations were performed as follows: All slices were analyzed and the slice adjacent to that of stem cell implantation was chosen. Two regions of interest (ROIs) were manually drawn over the ipsilateral hemisphere of stem cell implantation and the contralateral hemisphere (Supplementary Fig. S10a). Pixel intensity histograms were generated for each ROI in ImageJ (Supplementary Fig. S10b). A high signal intensity pixel at the crest of the normal distribution of the histogram (of the contralateral hemisphere of stem cell transplantation) was chosen as a threshold (Supplementary Fig. S10c). This threshold represented the maximum signal intensity in the absence of cell death. The ratio of the number of pixel intensities below the threshold in both hemispheres was then calculated. The value calculated at the first time-point was assigned an arbitrary 0 %. The values calculated at subsequent time-points were normalized relative to the first time-point and reported relative to this value (Supplementary Fig. S10c).

Supplementary Figures

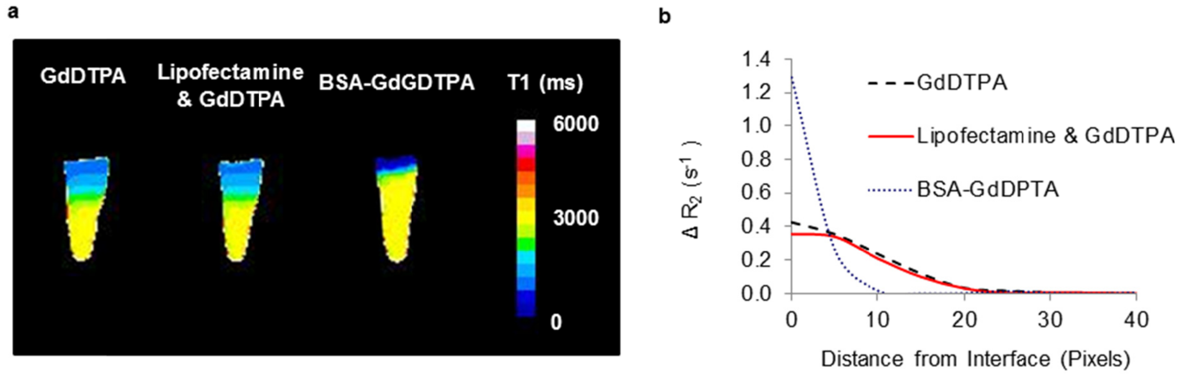

Figure S1. Diffusion Kinetics of GdDTPA solutions in the presence and absence of a transfection agent (Lipofectamine LTR). a) Respective T_1 maps of GdDTPA from three solutions, one hour post solution placement on 2% (w/v) agarose gel. The solutions studied included: GdDTPA in phosphate buffered saline (PBS); GdDTPA and lipofectamine LTR in PBS (5:1:25 v/v/v ratio); and an equimolar solution of bovine serum albumin-GdDTPA (BSA-GdDTPA) in PBS. BSA-GdDTPA was used here as a high molecular weight gadolinium chelate (negative diffusion control). **b)** GdDTPA diffusion kinetic profiles of the respective solutions, one hour post solution placement on 2% (w/v) agarose gel (n=3). The diffusion kinetic profiles of the GdDTPA solutions in the presence and absence of Lipofectamine LTR were similar, and different from that of BSA-GdDTPA, the high molecular weight control, with a slow diffusion rate.

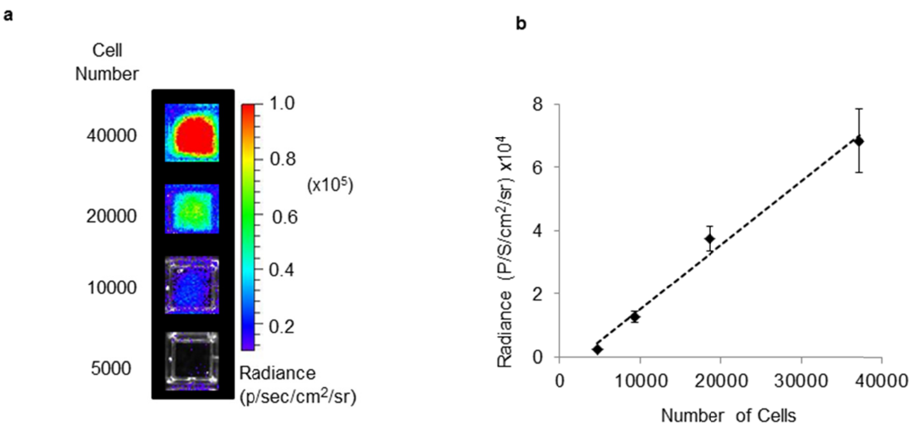

Figure S2. BLI of luciferase transduced cells. a) BLI radiance from varying numbers of luciferase-transduced cells. **b)** BLI radiance from varying numbers of luciferase-transduced cells shows a linear proportionality to the number of cells (n=2).

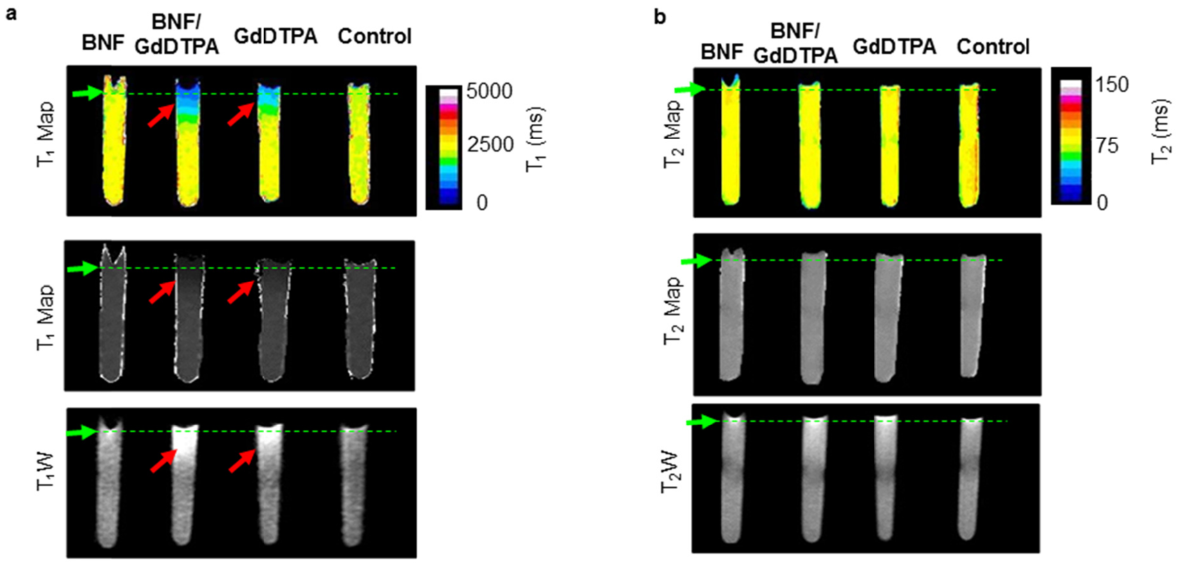

Figure S3. Phantom imaging of cell death detection. a) T_1 maps and corresponding T_1 images of 2% (w/v) agarose gel, on which were placed respective magnetically labelled dead cell samples (n=3). (↑) represents T_1 contrast enhancement in the vicinity of dead dual magnetically labelled cells. **b)** T_2 maps and corresponding T_2 images of 2% (w/v) agarose gel, on which were placed respective dead cell samples (n=3).

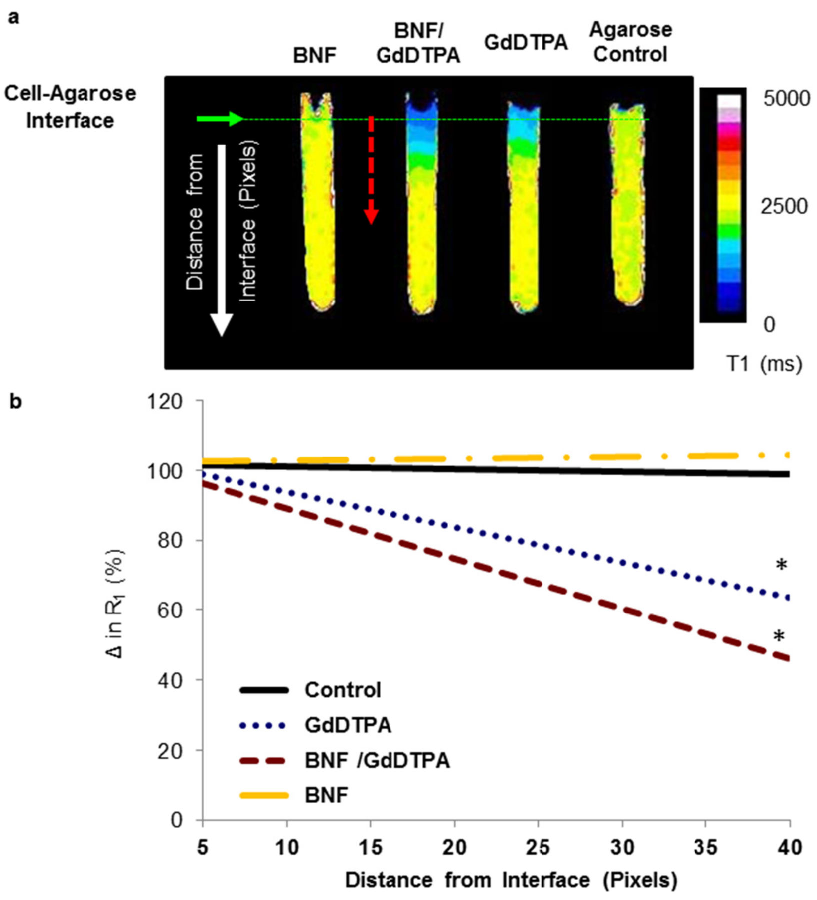

$$Normalized\ Change\ in\ Relaxivity = \left(\frac{Relaxivity\ X\ pixels\ from\ cell - agarose\ interface}{Relaxivity\ 5\ pixels\ from\ cell - agarose\ interface}\right) X\ 100\%$$

Figure S4. Cell Death Detection in Phantoms a) T_1 maps of agarose phantoms, topped with dead labelled cells (n=3). (green arrow) indicates cell-agarose interface, while (red dashed arrow) indicates measured distance along the tube. **b)** Normalized relaxation rate changes (mean ± s.d.), (%ΔR_1) along the agarose tube (n= 3; $P < 0.0001$). The statistics was done using a Kruskal–Wallis one-way analysis of variance, followed by a post-hoc Scheffé test based on the Wilcoxon-Mann-Whitney-test. All samples were technical replicates.

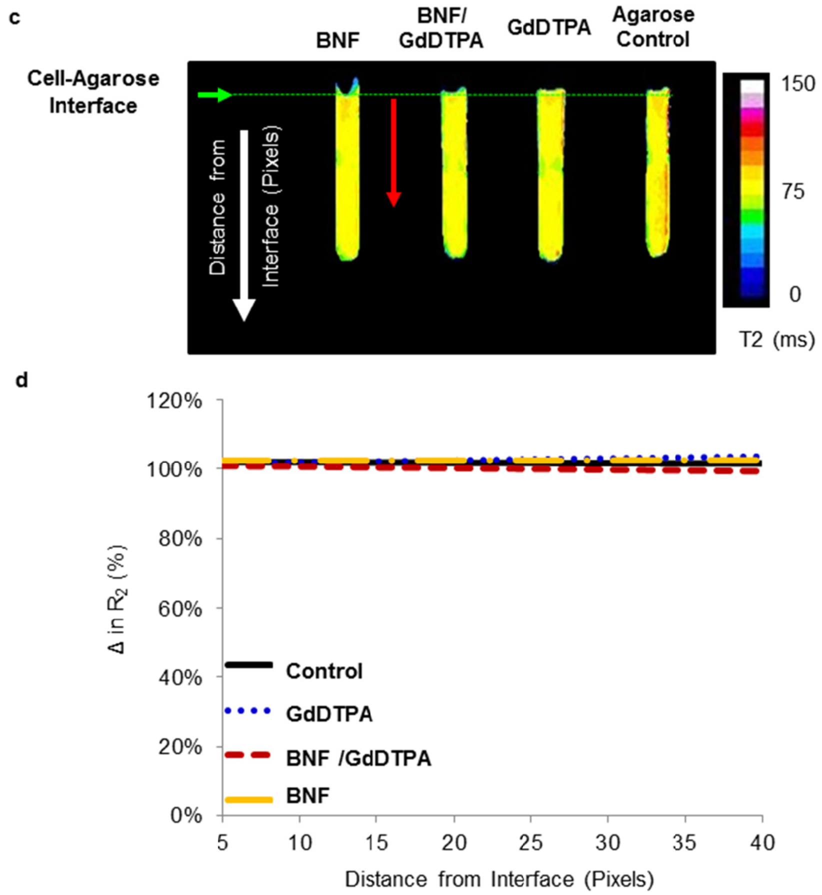

$$Normalized\ Change\ in\ Relaxation\ Rate$$

$$= \left(\frac{Relaxation\ rate\ X\ pixels\ from\ cell - agarose\ interface}{Relaxation\ rate\ 5\ pixels\ from\ cell - agarose\ interface}\right) X\ 100\%$$

Figure S4. Cell Death Detection in Phantoms c) T_2 maps of agarose phantoms, topped with dead labelled cells (n=3). (↑) indicates cell-agarose interface, while (↓) indicates measured distance along the tube. **d)** Normalized relaxation rate changes (mean ± s.d.), (%ΔR_2) along the agarose tube (n=3). All samples were technical replicates.

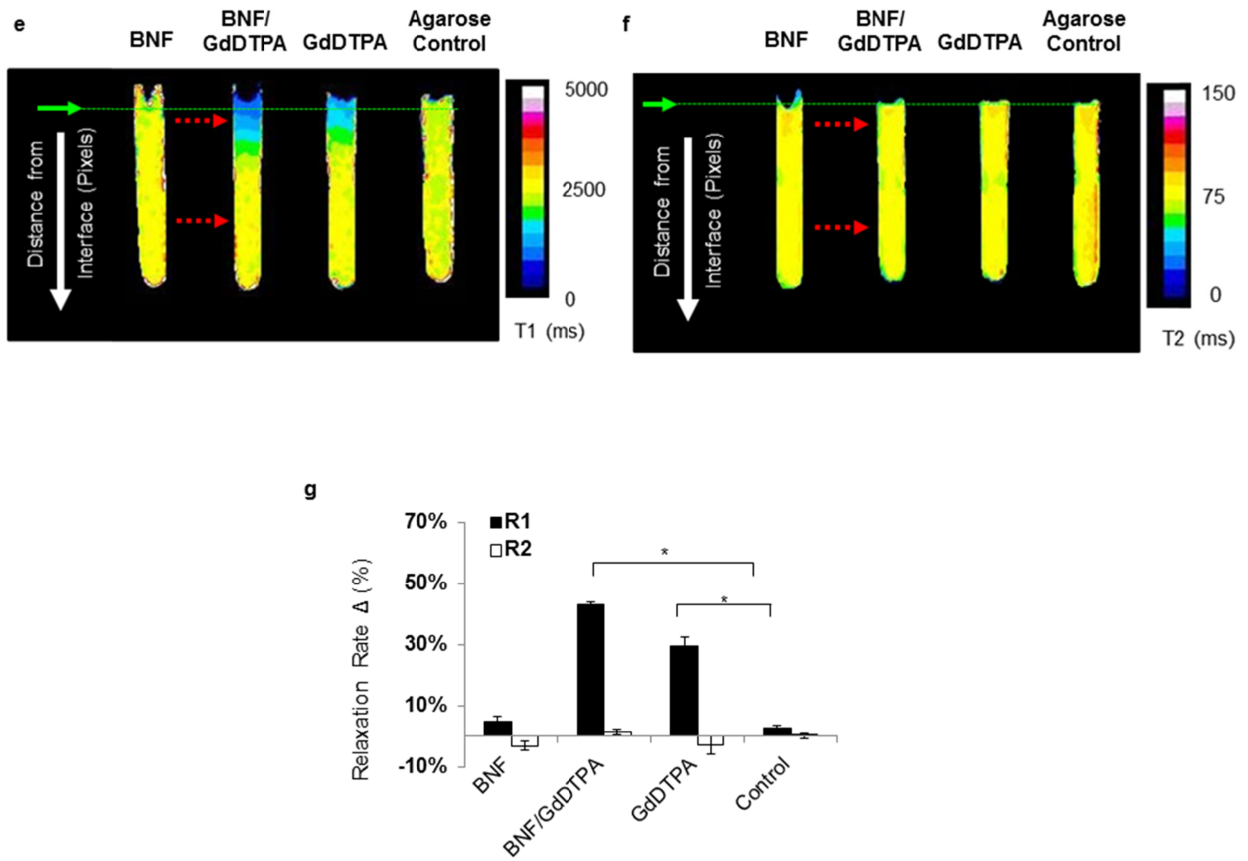

$$\begin{aligned} &Relaxation\ Rate\ Change \\ &\quad = (Normalized\ relaxation\ rate\ 10\ pixels\ from\ cell - agarose\ interface) \\ &\quad - (Normalized\ relaxation\ rate\ \ 90\ Pixels\ from\ cell - agarose\ interface) \end{aligned}$$

Figure S4. Cell Death Detection in Phantoms e) T_1 and T_2 maps of agarose phantoms, topped with dead labelled cells (n=3). ( ) indicates cell-agarose interface, while ( ) indicates measured pixels along the tube **f)** Normalized relaxation rate changes (%ΔR_2), along the agarose tube (n=3). **g)** Relaxivity changes (mean ± s.d.), ~10 pixels from the cell-agarose interface in the respective tubes shown in **c** (n=3, $P < 0.0001$). The statistics was done using a Kruskal–Wallis one-way analysis of variance, followed by a post-hoc Scheffé test based on the Wilcoxon-Mann-Whitney-test. All samples were technical replicates.

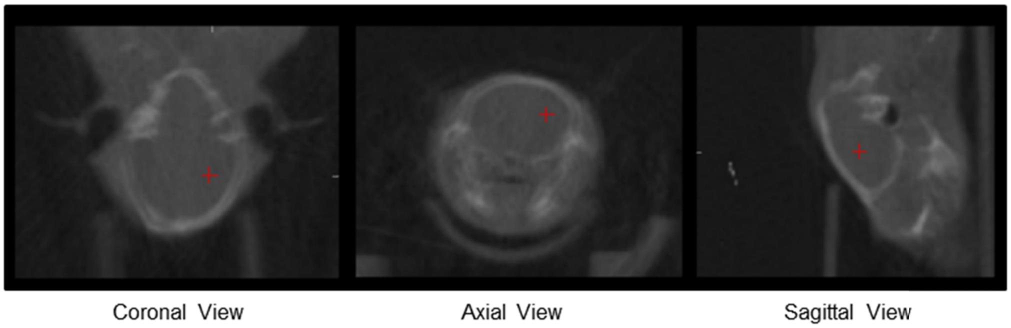

Figure S5. Induction of radiation-induced lesions in mice: Cone-beam CT acquisition geometry from a small animal radiation research platform (SARRP) shows a representative target irradiation beam from the: coronal; axial; and sagittal planes, respectively.

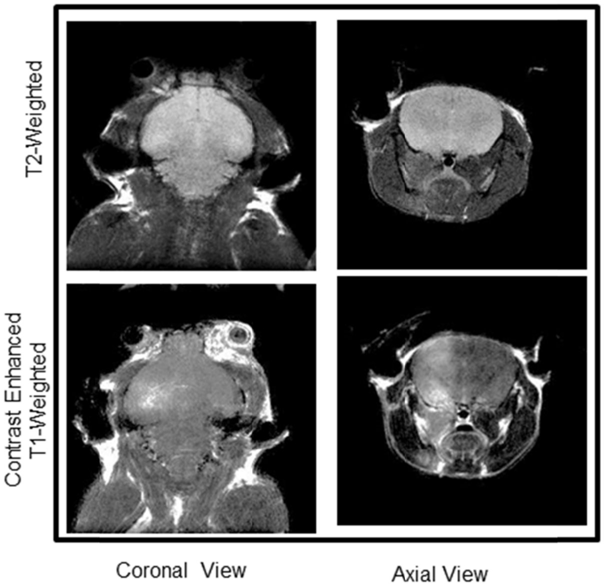

Figure S6. Radiation-induced brain injury in mice. T_2-weighted and contrast-enhanced, T_1-weighted MR images of a representative irradiated mouse brain, two weeks post irradiation.

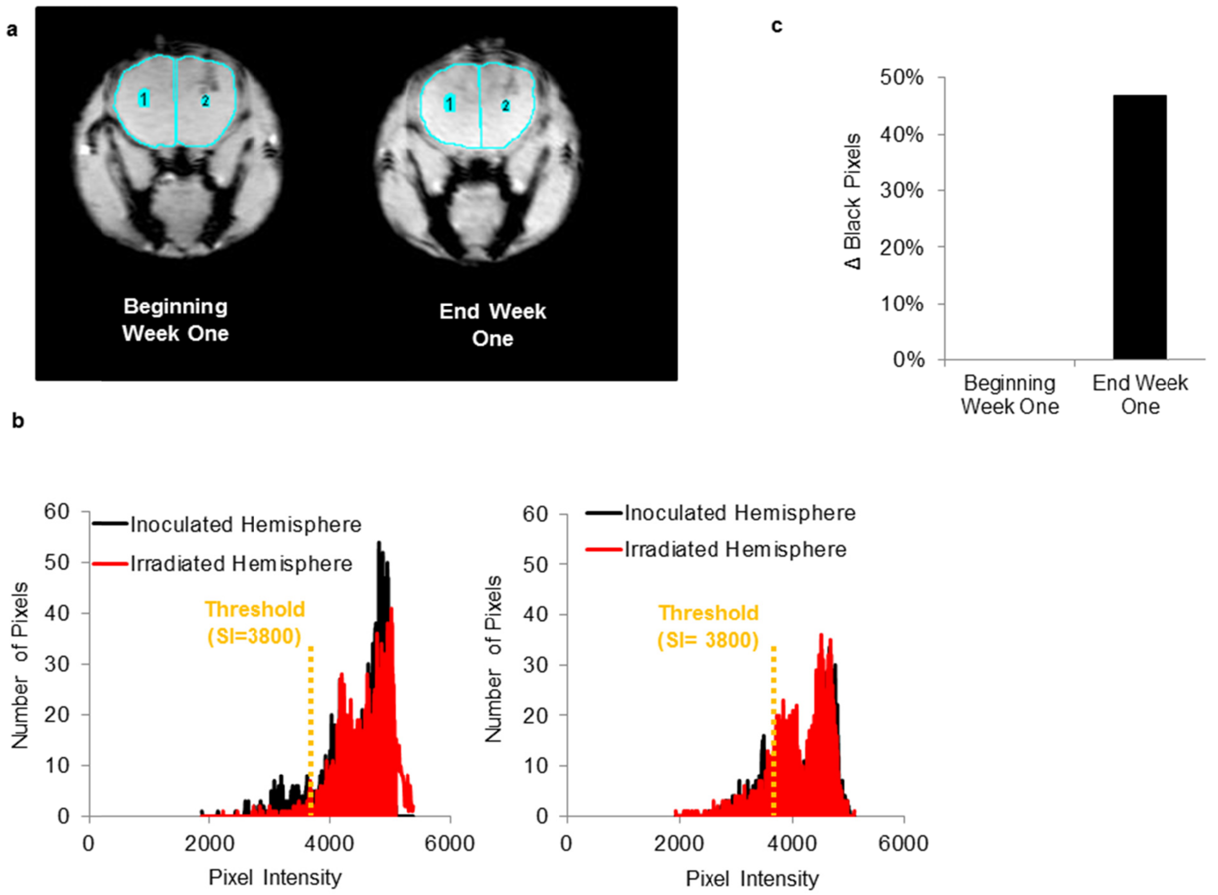

Figure S7. ***Black Pixel Analyses.*** **a)** Minimum signal intensity Z-projections of T_2*-weighted images of a representative immune-deficient mouse brain, generated from the slices of stem cell implantation. Two regions of interest (ROIs) were manually drawn over the contralateral hemisphere (ROI 1) and ipsilateral hemisphere (ROI 2) of stem cell implantation. **b)** Signal intensity histograms generated from both ROIs at the beginning and end of week one. A low signal intensity pixel was chosen as a threshold. **c)** The ratio of the number of low signal intensity pixels below the threshold in both hemispheres normalized using the ratio acquired at the beginning of week one (n=1).

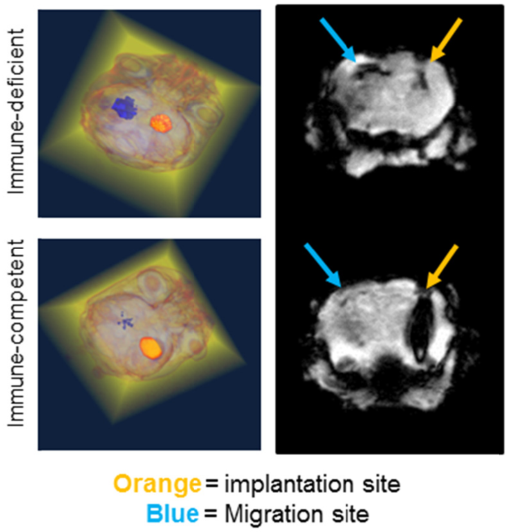

Figure S8. Cell Distribution c) Three-dimensional reconstruction of T_2^*-weighted MR images of brains from immune-deficient and immune-competent mice, and corresponding original and representative T_2^* slices from which the model was reconstructed. The images indicate cell distribution at both the implantation and radiation-induced lesion sites. (↑) represents the dual magnetically labelled cells lodged at the implantation site. (↑) represents the migrated dual magnetically labelled cells at the radiation-induced lesion sites.

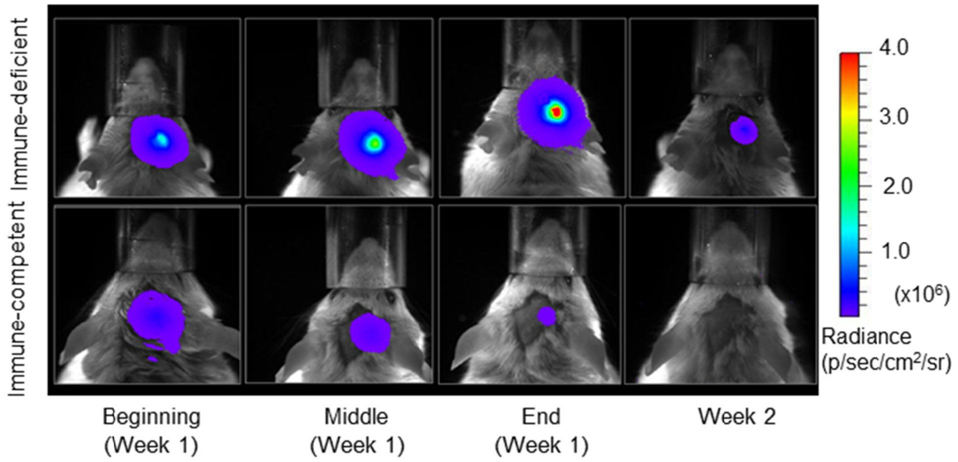

Figure S9. Cell death in immne-deficient mice at a later time point compared to immune-competent mice. Bioluminescence images of cell death in immune-deficient and immune-competent mice, respectively, over two weeks of stem cell transplantation

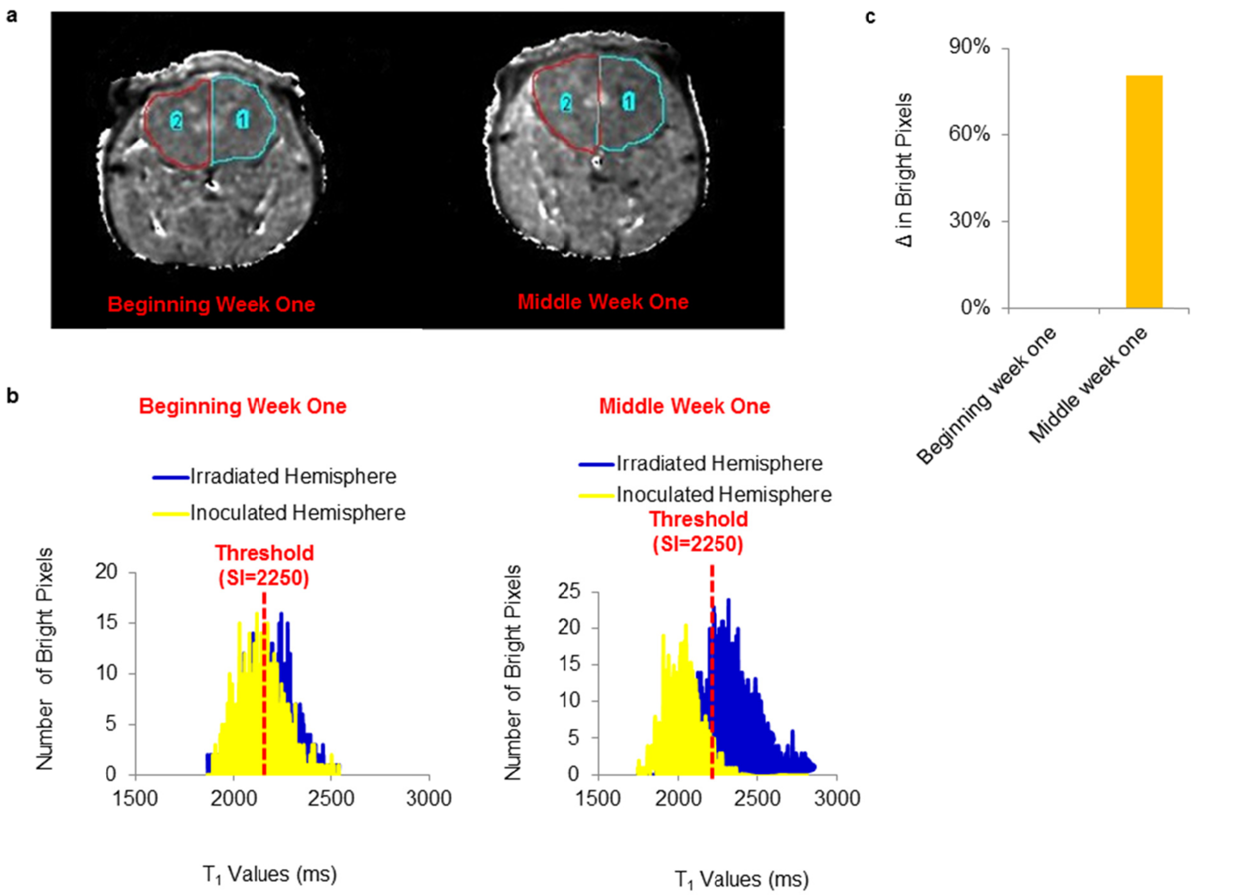

Figure S10. Bright Pixel Analyses. T_1-maps of the slice adjacent to that of stem cell implantation in a representative immune-competent mouse. Two regions of interest (ROIs) were manually drawn over the ipsilateral hemisphere (ROI 1) and contralateral hemisphere (ROI 2) of stem cell implantation. b) Pixel intensity histograms generated from both ROIs at the beginning and end of week one. A high signal intensity pixel was chosen as a threshold. c) The ratio of bright pixels (low T_1 values) below the threshold in both hemispheres normalized using the ratio acquired at the beginning of week one (n=1).

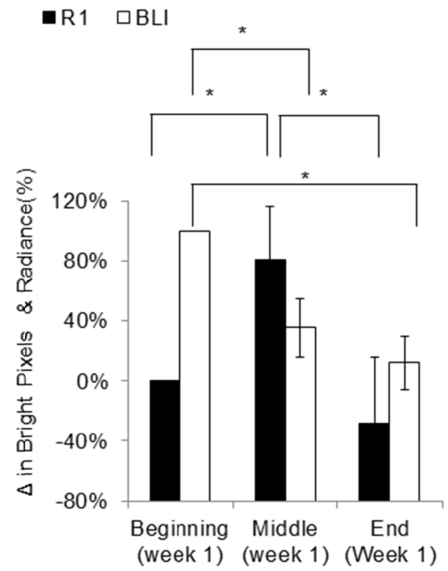

Figure S11. Comparison of T_1 contrast enhancement and BLI radiance decrease in immunocompetent mice following cell death. Quantitative T_1 contrast enhancement (mean ± s.d.) and BLI radiance (mean ± s.d.) in immunocompetent mice within week one of cell implantation. The statistics was assessed using a Kruskal–Wallis one-way analysis of variance, followed by a post-hoc individual comparison Scheffé test, based on the Wilcoxon-Mann-Whitney-test (n= 5; $P < 0.05$).
